# The Army Medical Museum and Library

**Published:** 1896-12

**Authors:** D. L. Huntingdon

**Affiliations:** Deputy Surgeon-General, U. S. A., In Charge of Army Medical Museum and Library.


ARTICLE II.

### THE ARMY MEDICAL MUSEUM AND LIBRARY.

BY DR. D. L. HUNTINGDON,
Deputy Surgeon-General, U. S. A.,
IN CHARGE OF ARMY MEDICAL MUSEUM AND LIBRARY.

**Read before the Union Meeting of the Maryland State and the Washington City Dental Societies, May 8, 1896.**

It is with much pleasure that I respond to the invitation of the chairman of your joint committee of arrangements to make a few remarks relative to the Library of the Surgeon-General's Office and Army Medical Museum, in connection with the resolution adopted by the thirty-sixth annual meeting of the American Dental Association, formally recognizing the Library of the Surgeon-General's Office and Army Medical Museum as the National Library and Museum of the dental profession of the United States.

In acknowledging the honor thus conferred upon us; I have, as librarian and officer in charge of the library and museum division of the Surgeon-General's Office, communicated with your chairman' stating that we shall be most happy to co-operate with your committee, and with the dental profession of the country, in forming a collection thoroughly illustrative of all matters pertaining to the subject of dentistry, and trust that, with an increasing knowledge of our facilities, a genuine interest may be manifested in carrying into execution a work so important to you and advantageous to ourselves.

It has, therefore, seemed proper that I should give you a very brief account of these institutions, which are so well known to the medical profession at home and abroad, as well as of their scope and capabilities, that you may feel assured of the wisdom of your action in selecting them as the national repository for all matters pertaining to your specialty,

Previous to the late Civil War, the Library of the Surgeon-General's Office was composed of a few hundred books on medicine and surgery and collateral branches; very much such a collection as might naturally collect in the office of the chief of an important bureau.

Few additions were made to this collection until the year 1865, when Dr. John S. Billings, then an assistant surgeon of the army, was detailed to take charge of it. Dr. Billings at once recognized the value of the opportunity thus presented for laying the foundation of a great national medical library. As a result of his energy, knowledge and judgment, we have to-day the magnificent Library of the Surgeon-General's Office.

In the earlier years of its growth this collection was placed in the old Ford Theatre building, which, after the tragic assassination of President Lincoln, was purchased by the Government and turned over to the medical department of the army for the use of the Library and Medical Museum.

Aided by liberal appropriations from Congress and directed by the energy and perseverance of Dr. Billings, the growth of the library have been phenomenal. In a few years its constantly increasing size and value, as well as the continual menance of destruction or damage by fire in the old and exposed building, made it imperative to provide secure and ample accommodations elsewhere.

In 1866, Congress passed a bill appropriating the requisite funds for the erection of a spacious fire-proof building. This building, located near National Museum and Smithsonian Institution, was completed and ocpupied in 1887. From that date to the present time its growth has been continuous, until it has now come to be acknowledged "the most complete library in the world," containing on its shelves to-day about one hundred and twenty thousand volumes of bound books and about two hundred thousand pamphlets, together with a most valuable collection of atlases of plates and engravings illustrating anatomy, surgery, physiology, and obstetrics.

In its general scope the library is intended to cover the entire field of medical and surgical literature, making it possible for the student to avail himself of the writings and teachings of all countries and all times, with the least possible loss of time and labor. While intended mainly as a reference library, a certain latitude is observed by the management with a view to making it as useful as possible to the medical profession, and especially to students and writers, by loaning to well-established medical societies and libraries of prominent medical colleges such books as may be called for, upon the condition that these bodies will guarantee the care and return of books loaned, and be responsible for all losses and damage which may occur.

Rare and valuable books and manuscripts, plates, unbound books, and theses, whose loss cannot be easily replaced, are not loaned.

Connected with the library is a reading-room, where all the current journals and periodicals are kept on file for the convenience of those desiring to consult them. To those making special studies in any line of the profession we are prepared to afford all possible facilities.

The annual list of journals and periodicals subscribed for and received at the library amounts to over eleven hundred, and embraces all subjects pertaining to the literature of medicine at home and abroad. The machinery for the increase of the library is complete. Through agents in all the principal cities in the world, we are constantly receiving not only the current and latest medical literature, but frequently have the opportunity to secure rare and valuable works of past centuries. The appreciation in which this library is held is best evidenced by the frequent and large donations of books and pamphlets from every source, among which are many duplicates of works in our posession, which afford us the means of obtaining desirable additions through exchange.

The library receives its main support from the liberality of Congress. Since the year 1867 up to the present time, the appropriations of this body for the support of the library have averaged about seven thousand dollars annually, which enables us, with a fair degree of certainty, to keep our journal files complete and make very considerable additions of new books, as well as insuring a small annual amount for the purchase of old and rare books and curiosities of medical literature. The practical value and utility of this collection is proven daily by the members of the profession, who make regular use of the facilities offered—by the interest shown by medical men from the United States and from abroad. The reading room is constantly occupied by readers, students, and investigators. This vast wealth of medical literature is made accessible to the profession through the medium of the Index Catalogue, which is at present comprised of sixteen large octavo volumes of about one thousand pages each. This work was begun in 1879, and has progressed continuously at the rate of one volume per year. The catalogue is arranged by authors and subjects, and may fairly be considered "as practically an index to all the medical literature of the world up to the end of this century."

The large accessions of material since the publication of the volumes are carefully carded, and will appear in a second series of volumes, the first of which is now in the course of preparation, to be followed by as many more as may be required and dependent upon the continued liberality of Congress. A comparatively small edition of these volumes is printed, and is distributed mainly to medical libraries, medical colleges, and to certain of the larger public libraries of the United States and Europe.

By reference to this Index Catalogue, Vol. III, 1882, under the proper heading of "Dental," "Dentists," and "Dentistry," you will find not less than six closely printed pages, or thirteen columns, referring to works and journals under these captions. Since the publication of this volume the additions to the library on these subjects have been very large, and will be printed in their proper volume of the second series.

Of journals and periodicals pertaining to dentistry, our list embraces all of the best throughout the world, and from our agents we are continually receiving the latest works on the subject.

The Army Medical Museum, occupying the eastern wing of the building already alluded to, is also of comparatively recent origin, and owes its existence to ex-Surgeon-General W. A. Hammond, who, in 1862, issued a circular stating that it was "proposed to establish in Washington an Army Medical Museum," and directing medical officers of the army "diligently to collect and forward to the office of the Surgeon-General all specimens of morbid anatomy, surgical and medical, which may be regarded as valuable; together with projectiles and foreign bodies removed, and such other matters as may prove of interest in the study of military medicine and surgery." The response to this circular was immediate and gratifying. From the large general hospitals and from the battle-fields, as well as from private sources, came extensive contributions to this enterprise, so that a catalogue, printed in 1866, showed a collection of 7,716 specimens of all kinds. To the administrative ability and personal interest of Surgeon-General J. K. Barnes we are indebted for the successful establishment of this museum on a permanent basis. Succeeding Surgeons-General have manifested similar interest in perpetuating and fostering its growth.

To Drs. John H. Brinton, J. J. Woodward, George A. Otis, J. S. Billings and others are due its scientific value and importance, each of these gentlemen having given time, care, and learning in building it up to its present condition.

On the completion of the new library and museum building, this collection was transferred from the old Ford's Theatre building, with the library, to its present spacious and secure quarters.

At the present time the collection contains over 33,702 specimens of all kinds, which may be divided as follows:

Pathological, 12,164; human anatomy, (normal), 4,470 comparative anatomy, 1.717; microscopical specimens, 12,157; medals, 1,205; instruments and apparatus, 1,044; microscopes (showing evolution of the instrument), 188; miscellaneous, 457.

The annual addition of specimens in all the above lines is large and constantly increasing.

Connected with the museum are extensive pathological and bacteriological laboratories which serve as important feeders to the main collection. * * *

It would be useless and tedious to enter upon a detailed description of this collection; it must suffice to say that as a storehouse of the results of military medicine and surgery of thirty years ago it is probably the most unique and valuable collection in the world, affording a curious and interesting comparative study of methods and treatment of that period with the advanced views and practice of to day.

But this museum is not solely a receptacle for the results of the work of the past; it has kept well abreast of the times, and presents in its later accumulation the evidences of the advance of scientific medicine and surgery. Departing from its original limitation to military medicine and surgery, our museum has greatly extended its scope, and now includes the departments of human anatomy and osteology, physiology, embryology, pathology, and anthropology, with illustrations of the methods of research connected with all the branches of practical medicine.

As will be seen by referring to the classification of specimens, we have made the largest use of photography and micro-photography in illustrating tissues and structures of the human body. Within our miscellaneous department are included models for the study of veterinary medicine and surgery, and some space is devoted to prosthetic apparatus, and to instruments and appliances used in the several departments of medicine and surgery; in fact, we desire through this museum to effect through object teaching that which is secured in the library by written works and treatises, viz, a thorough illustration of the science of medicine as it is found at the present day.

In this very brief description of the museum and its scope I have hardly done more than give to it a "certificate of character, for I feel that I must not encroach too much upon the time which has been so kindly alloted to me, and I now desire to come to the point of what I have to say relative to dentistry.

Our museum is not so well furnished with material relating to your profession as is our library. The collection of books and journals is a part of the library management, and it is our care to see that the several departments of medicine are well and harmoniously rounded out.

Books and journals we can obtain by purchase; *specimens pertaining to your daily work, illustrative of normal and morbid dentition, and of diseases of the oral cavity, we cannot make, nor can we always buy;* therefore I appeal to you, individually and collectively, to manifest not only sympathy in the proposed movement, but to give evidence of a practical interest by contributions, that we may be able to erect within our hall an exhibit of dentistry which shall be worthy of your profession.

By gifts and by limited purchases we have already what may be called a nucleus for such an exhibit. We have sufficient space and all the facilities necessary for the purpose, and all we ask is the material. Look through your private collections and separate such specimens as, in your opinion, would be appreciated by your dental brethren, if placed where they could be seen and studied. Throughout this country there must be thousands of such specimens which, if collected in one place, would form an unrivaled exhibit, to say nothing of their value as means of instruction to the student and investigator.

You may ask me what we especially desire for this exhibit? I would suggest casts, photographs, and specimens of normal, morbid, and anomalous dentition, of diseases of the maxillæ and oral cavity; photographs and casts of surgical operations, prosthetic apparatus of all kinds used in your work, new instruments, and specimens of mechanical work, and any miscellaneous material which may lend an interest to the subject of dentistry. All specimens intended for the museum should be accurately labeled and accompanied by a concise description and history of the case, when possible.

Do not be deterred from making contributions on the ground that we probably have many such specimens as the one proposed to be sent. Of all things we need duplicates, not only for inter-comparison, but for the purposes of exchange with other institutions, whereby both our own and others are benefitted.

It is not necessary that you should attempt to mount your specimens; send them to us as they are, and they will be prepared and mounted by our pathologist.

Neither is it necessary that freight or express charges be prepaid; address your contributions to the Curator of the Army Medical Museum, Washington, D. C., and they will be received and cared for.

So soon as the number of contributions relating to the subject of dentistry will warrant, it is the intention of the management of the Museum to collect them together into one department where they can be exhibited and studied to the best advantage; it is also proposed to appropriate a certain amount annually for the purchase of models, preparations and appliances pertaining to dentistry.

In common with medicine and surgery and their collateral branches, dentistry has made great strides of advancement during the last quarter-century. Your methods of instruction and training have constantly improved; your text-books and treatises are becoming fuller and more complete; your journal literature abounds in thoughtful, live articles, showing a just appreciation of the demands of the age, as well as of the necessity for basing the superstructure of your specialty on the broad foundation of a thorough knowledge of general anatomy, physiology, and pathology; your professional future promises still greater advance and progress.

Now, in what way can our Museum contribute to your professional benefit? I would answer that such a collection is an educator; a means for the diffusion of useful special knowledge. Within our walls you will find a record of what has been done and what is being done. It is the function of the Museum to preserve this record for all time. By the study of its collection you will be enabled to make comparison of the methods of practice and the modes of thought of your professional co-workers; to enlarge your knowledge of normal and abnormal and anomalous development; to study pathology in forms which may not have previously met your eye; finally, to enlarge generally your powers of observation and widen your views.

It has been a fundamental idea in the origin and development of this Museum, and especially with my predecessor, Dr. Billings, that this collection should be not merely a repository of the curious and interesting in medical science, but also a school for the demonstration of methods of research for the benefit of investigators and teachers.

It is for this purpose that I ask your interest and action in carrying out the spirit and intent of the resolution of the American Dental Association, by assisting us to build up a creditable exhibit of your specialty, which in time shall be an important and invaluable aid to your students and practitioners.

As I have before said, you shall have the cordial cooperation of our management in making this department eoual to any other of the Museum; and, in closing, I heartily invite you, during your session in this city, to visit both the Library and the Museum, and see for yourselves what can be but imperfectly described in so limited a time.

---

ARTICLE III.

### NOVEL OPERATION ON THE EYE.

